# Quantum Key Distribution: Modeling and Simulation through BB84 Protocol Using Python3

**DOI:** 10.3390/s22166284

**Published:** 2022-08-21

**Authors:** Akwasi Adu-Kyere, Ethiopia Nigussie, Jouni Isoaho

**Affiliations:** Department of Computing, University of Turku, Vesilinnatie 5, 20500 Turku, Finland

**Keywords:** quantum key distribution, quantum mechanics laws, cybersecurity, eavesdropper detection

## Abstract

Autonomous “Things” is becoming the future trend as the role, and responsibility of IoT keep diversifying. Its applicability and deployment need to re-stand technological advancement. The versatile security interaction between IoTs in human-to-machine and machine-to-machine must also endure mathematical and computational cryptographic attack intricacies. Quantum cryptography uses the laws of quantum mechanics to generate a secure key by manipulating light properties for secure end-to-end communication. We present a proof-of-principle via a communication architecture model and implementation to simulate these laws of nature. The model relies on the BB84 quantum key distribution (QKD) protocol with two scenarios, without and with the presence of an eavesdropper via the interception-resend attack model from a theoretical, methodological, and practical perspective. The proposed simulation initiates communication over a quantum channel for polarized photon transmission after a pre-agreed configuration over a Classic Channel with parameters. Simulation implementation results confirm that the presence of an eavesdropper is detectable during key generation due to Heisenberg’s uncertainty and no-cloning principles. An eavesdropper has a 0.5 probability of guessing transmission qubit and 0.25 for the polarization state. During simulation re-iterations, a base-mismatch process discarded about 50 percent of the total initial key bits with an Error threshold of 0.11 percent.

## 1. Introduction

An increasing body of literature recognizes both the importance and emergence of quantum computers [1,2]. The quantum principles on which these future computers will rely have a crucial role in today’s communication security and have received considerable attention recently [3]. These principles and properties are becoming a key instrument in how current security infrastructural design may need to adapt towards a post-quantum era. Different platforms and service categories utilize these diverse security infrastructures to secure communications and share data through cryptographic mechanisms. The purpose is to ensure information risk management despite attacks on communication protocol stacks. Both researchers and market analysts commonly suggest that quantum technologies such as QKD will be essential for a wide range of Internet communication technologies based on the current market demand [4]. This relationship between quantum and classic cryptography will likely influence several sectors in the coming years.

The Internet of Things (IoT) plays an increasing role in sectors such as cyber–physical systems and autonomous systems, which extend versatility to human-to-machine and machine-to-machine, vehicle-to-things, and vehicle-to-Internet (V2I) interactions [5]. Today, solving relevant and on-demand technological challenges such as data retrieval, automation, analysis, machine learning (ML), and monitoring processes in intelligent environments is achievable across multiple cloud platforms through several Internet services. These services include data collection and sharing supported by numerous hardware-to-service nodes on heterogeneous devices over current classic communication channels. IoT security, challenges, and importance for these related service categories depend on cryptographic technologies that utilize symmetric and asymmetric algorithms. However, the current state-of-the-art key distribution and management processes face constraints and challenges such as managing numerous encryption keys, threats from malicious insiders and intruders, data accessibility by non-authorized users, governance, and application support, while dependent on the communication channels for communication secrecy. They depend on mathematical difficulty and computational complexities [6,7] compared to quantum technologies and cannot detect eavesdropping. This inability and dependency variation may allow malicious intruders and insiders to use clever and efficient ways to actively or passively manipulate and complicate secure secret key transmission and distribution. It can also influence the end-to-end trust in an ecosystem with differential security levels.

For example, a typical asymmetric (public key) cryptographic system has three components. These components are the message (plain-text) to be encrypted, denoted as *M*, the key used for the encryption *K*, and finally, the output (cipher-text), which is the encrypted message *C*, as shown in the figure below. Two keys are utilized for encryption and decryption [8]. One of the keys is public (encryption key), while the other is secret or private (decryption key). The publicly available is for anyone who wishes to communicate securely with the owner and holder of the private key.

**Figure 1:**
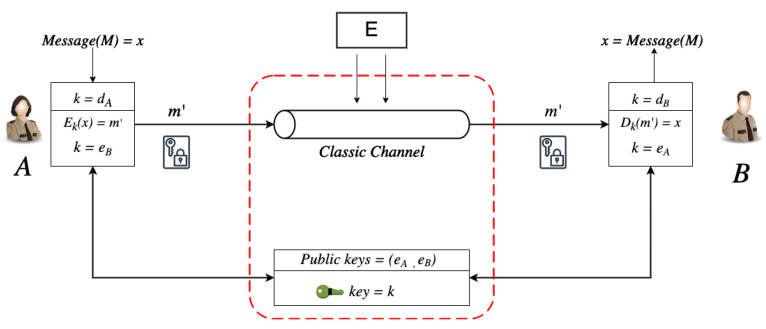
This figure represents a basic public key cryptosystem.

The decryption of the cipher-text uses the second part of the key as $D_{d}B\left(m^{\prime }\right)=\left(x\right)$. Figure 1 illustrates this process by using two parties, A and B. Both Party A and B have a secret key and a private key ($d_{A},e_{A}$) and ($d_{B},e_{B}$), respectively. Assume Party A wants to send a message M=(x) to Party B by using Party B’s public key ($e_{B}$) for the encryption ($E_{e}B\left(x\right)=\left(m^{\prime }\right)$). Public key infrastructures such as the Rivest–Shamir–Adleman (RSA) algorithms rely on the inability to factorize larger integers of the form n=PQ effectively [9] in a realistic time (polynomial time) [10], hence applying computational complexity and mathematical difficulty to increase or decrease the security robustness [6,11,12,13].

This work studies and demonstrates QKD use for secure cryptographic key distribution over a classic communication channel. It focuses on implementing a standard de facto BB84 protocol in a simulation model design. The contributions of this work are:

1. Design of a communication architecture model that takes advantage of quantum cryptography for enabling secure communication;
2. Implementation and simulation of the BB84 protocol in python3;
3. Analysis of QKD efficacy for secure communication.

The first section is a theoretical overview of quantum cryptography (QC) and commonly used terminologies in this paper. The next section describes the communication architecture model, followed by its implementation. Further characterization of the architectural model illustrates our simulation for the no-cloning theorem and uncertainty principle with and without an eavesdropper. Finally, the research findings focus on QKD’s importance.

## 2. Overview of Quantum Cryptography

A century ago, Steve Wiesner’s paper *Conjugate Coding* considerably ignited quantum cryptography’s realization [14] after a series of contributing events. For example, Max Planck discovered Planck’s constant by finding ways to explain his glowing light filament observation [15]. Einstein’s 1905 prediction and Sir Isaac Newton interpreted light as a wave and not just an energy source with millions of elementary particles [16]. Each particle’s discrete quantity of energy is proportional in magnitude to the source frequency emission and transformation of light. This development, later on, led to photons through Arthur Compton’s work in 1923 [17]. However, the 20th Century [12] evidences these contributions in current quantum cryptographic popularity and the evolution of advancements in the reality of quantum principles and concepts.

Today, the *“science of secrets”* [18] as we now know through photon–quanta energy manipulation has benefited a wide range of technologies such as QKD. It is now among one of the fully developed and heaviest research focus areas in quantum informatics [19,20]. This advancement is partly due to the prospects of quantum computing and classic cryptographic systems’ shortcomings benefiting QKD’s trends. QKD’s cryptosystem basis and construction reveal a guarantee of secrecy explicitly attributed to the laws of nature in quantum mechanics [21]. It is a mechanism for agreeing on secret shared keys between remote parties [22] to ensure tamper-proof shared keys via alerting the original parties if tampered with during transmission by an adversary.

**Figure 2:**
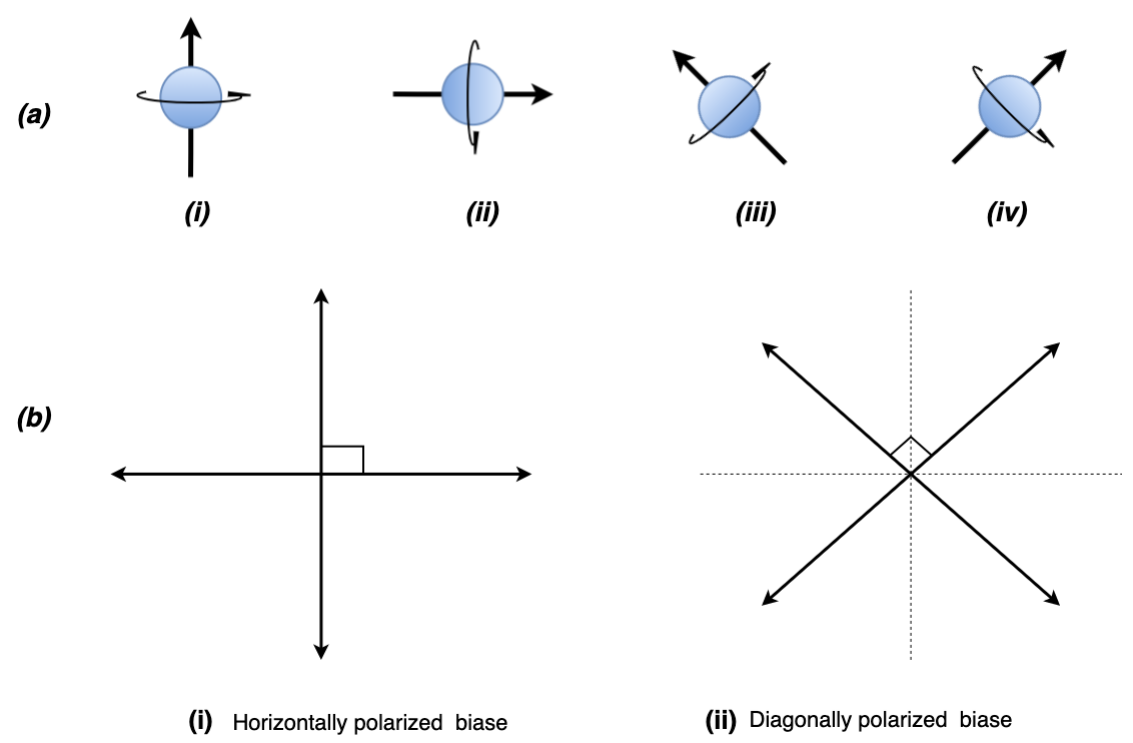
This is a figure representing the spinning direction of the qubit and its quantum states at a specific time: (**a**) Shows the four states of polarization qubits. (**b**) (i,ii) illustrate the two types of polarization for encoding purposes in quantum cryptography only detectable by the correct photon filter.

Now, let us clarify a few necessary terms used throughout this paper. A *qubit* is a classic bit in a quantum system. Qubits in a quantum domain spin continuously in a direction dependent on the propagating source, as shown in Figure 2. This spinning property is the quantum state [13], referring to a condition of an entity being differentiable from others of its kind at a specific instance. Determining this state requires measurement, which could be through observation. However, measuring a quantum state introduces a disturbance that irretrievably changes the state, leading to our first core principle, *Heisenberg’s uncertainty principle* [23]. It states that measuring a photon’s quantum state is impossible without introducing a disturbance within a quantum system. This uncertainty principle implies that a change in a quantum state is the direction in which a qubit spins at any particular time, shown in Figure 2, prior to measurement. It refers to this behavior as an unknown state, which is also the superposition theorem [24]. It is a property of a qubit and entanglement, where two or more qubits correlatively spin in a direction within a quantum system [25]. If the spinning direction of a single qubit is known, then this spinning direction can help determine multiple qubits’ directions. Hence, quantum bits for information transference from one point to another are restricted or induced to a defined pattern or direction for message encoding in polarization. They are horizontally or orthogonally biased before transmission over a protocol. A *protocol* is a systematic [26] and recommended set of procedures that officially govern how a specific activity’s internal operation occurs for profitable utilization. Quantum protocols share basic foundational principles, even though some specific characteristics and properties are unique to some protocols.

Based on the previously mentioned uncertainty property and characteristics, there would be challenges whenever copying a polarized photon because the quantum state of that specific photon is unknown. This leads to our final core principle: the *No-Cloning theorem*. This theorem relies on Wootters and Zurek’s no-cloning theorem in 1982, which states that the copying of a polarized photon is impossible due to the unknown quantum state of that specific photon [27]. Another aspect of this definition is that cloning a specific photon requires measurement parameters, including obtaining the quadrature component [28], which accurately represents the clone. However, this principle breaches the no-cloning theorem and is no longer a clone of that polarized photon.

In light of the uncertainty theorem, an eavesdropper has a probability of 0.5 of guessing the currently encoded qubit and 1/n chances for the polarized quantum states, where n is the amount of the existing state in that quantum system. Figure 2b illustrates this by showing that at any point in time, a qubit could be horizontally biased or octagonally biased with the probability existing in Figure 2a. For example, in a typical quantum cryptographic communication, a polarized photon’s direction of $0^{{}^{\circ}}$ and $45^{{}^{\circ}}$ may represent |0〉 as 0bit while $90^{{}^{\circ}}$ and $135^{{}^{\circ}}$ represent |1〉 as 1bit. Only either horizontal or orthogonal bias is detectable by the correct photon filter. It is a one-way operation in quantum cryptography. Significantly, photons detected by the photon filter or detector upon impact are not reconstructable. Moreover, undetected photons also suffer the same fate. Therefore, assume a photon filter detecting three photons polarized in the following ways, $(0^{{}^{\circ}}$ or $45^{{}^{\circ}})$, $(90^{{}^{\circ}}$ or $135^{{}^{\circ}})$, $(90^{{}^{\circ}}$ or $135^{{}^{\circ}})$, or $(0^{{}^{\circ}}$ or $45^{{}^{\circ}}$), would be encoded in a classical bit equivalent of a 1001 bit representation.

### Quantum Attacks

The principles of quantum cryptography relying on the laws of quantum mechanics for generating a secure key via manipulating light’s properties for secure end-to-end communication is theoreticallysound [29,30,31]. The versatile security interaction between “Things”, such as human-to-machine and machine-to-machine, currently benefits from this advancement worldwide through communication architectural quantum networks, a promise yet to spread across countless practical applications even with positive trends with technological evolutional advancements in their early stages. However, this promise and technological paradigm of this quantum regime have unprecedented challenges related to conceptualizing and interpreting quantum principles from theoretical to fully functional, practical quantum systems. These are noticeable technical imperfections, impeding physical barriers in coherent pulse generation, oscillators, interferometers, synchronizations, channel noise, and auto-compensating optical communication causalities. Even worse, these challenges cannot be generalized but have a moderate figure of merit on differentiable QKD vendor systems.

Quantum experimental hacking and exploitation attacks take advantage of the essential nature of light’s property as a wave other than packets of energy quanta, which requires an indistinguishable phase difference via introducing instability or interference in its hardware modeling nodes and devices. These practical non-uniformities attributed to attack causalities can also be extended to quantum protocol drawbacks and environmental influence. Today, quantum error rate (QBER) stemming from the error correction in a quantum key distribution systems (QKDs) has been a contributing factor as an attack vector for influencing the coherence photon state [32] source. Stimulated emission, which approximates perfect quantum cloning [33], parametric optical amplification, and the bunching properties of light fields [11] can be considered post-quantum polarization cloners in contrast to the no-cloning theorem. The process assumes a perfect cloning device capable of cloning and maintaining all the characteristics and properties of that specific polarized photon, even though they are affected by the fidelity of the equipment used. Relative attacks such as beam-splitting [34,35,36] have been a platform for daisy chaining other exploratory exploitations, such as calibration attacks [37,38], side-channel attacks [39,40,41], which extend wavelength manipulation [42,43] with a similar profile attack, such as detector-device-independent [44], denial of service attack [45,46], intercept-resend attack [47], and Trojan horse attack [48].

## 3. Related Work

The literature on the evolution of QKD has highlighted several advantages and disadvantages concerning possible attack scenarios and perceived weaknesses [49]. Different theories exist in the literature stemming from variations of the original BB84 protocol of Bennett–Brassard. More recently, attention has focused on the frameworks, algorithms, platforms, and software for simulating different experimental concepts and ideas [50,51,52,53].

Using the simulation approach, researchers can balance cost, convenience, and other factors that are complex to maneuver with hardware. A considerable amount of published literature has been on QKD simulation with this outcome. Some examples of these studies and research include Omer et al. [54], who simulated a QKD process based on the BB84 protocol. The core part of their simulation was written in Visual C sharp. Buhari et al. [55] used the OptiSystem platform. Antje Kohnle and Aluna Rizzoli [56] used either polarized photons or spin 1/2 particles as physical realizations. Chatterjee et al. [57] also simulated QKD based on the B92 protocol. Mogos [58] focused on two cases: with and without cyber-attacks using C plus-plus (C++). Shajahan, Rimitha, Nair, and Suchithra S. [59] explained how a networking scenario could exploit pure laws in physics through QKD simulation inside a classical communication channel. Khan et al. [7] presented an in-depth security analysis on QKD protocols encompassing theoretical assertions to practical implementation factors. Anuj Sethia and Anindita Banerjee [60] simulated a practical model implementation of differential phase shift (DPS) QKD with a toolkit based on Simulink and MATLAB. Kashyap and M. Ramachandra [61] and Mina Mihai-Zico and Simion Emil [62] simulated QKD in the Qiskit library of Python. Fan-Yuan et al. [63] simulated using a single-photon and Hong–Ou–Mandel interference optical units.

These works share key features that are consistent with the literature and the theoretical results. In contrast and as an extension to previous work, some analytical and simulation works did not clearly state the error threshold bound limit they were working with. There was an insufficient comparison between scenarios with and without the presence of an eavesdropper. In some instances, the simulations were by example rather than by modeling, making it complicated to compare the initial and final parameters.

## 4. Simulation Architectural Model and Implementation

**Figure 3:**
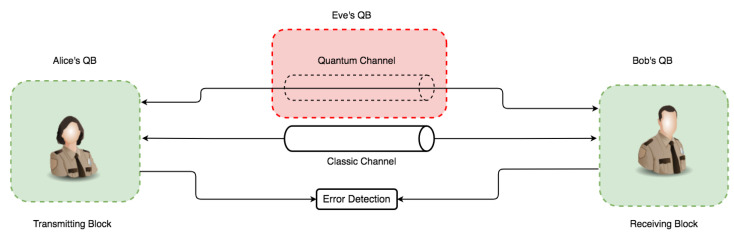
This is a figure representing the base overview of the simulation concept for subsequent simulation iterations.

Indeed, the possibility of intercepting a quantum transmission via a quantum channel is through disturbance. It is also clear that such activities are detectable via the quantum protocol’s error rate and eavesdropper presence. With these already-established core principles, in combination with the mathematical proof that it is impossible to decode a random one-shot key with an equal key and message length [64], an efficient QKD secure key distribution guarantees absolute secrecy between parties. To establish our proof-of-concept on the above characteristics, the communication architecture model in Figure 3 illustrates this in an overall conceptual simulation model with three main components called quantum blocks (QBs). The QBs represent the transmitter (Alice’s QB), the receiver (Bob’s QB), and the Eavesdropper block (Eve’s QB) as non-authorized access to the quantum channel.

**Figure 4:**
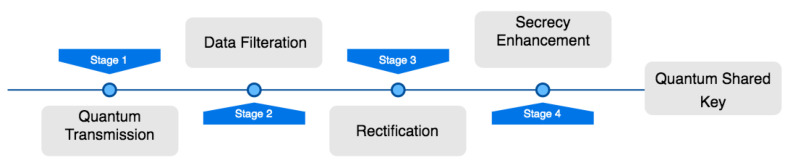
Main simulation procedure.

The overall simulation procedure in Figure 4 describes photon detectors filtering a polarized photon transported in quantum transmission and then rectified by the transmitting parties through bit comparisons, the error detection rate, and error correction. The output is optimized to enhance the security, enabling the total quantum shared key to meet the exact security requirements through a series of privacy amplification processes.

### 4.1. Architectural Model

**Figure 5:**
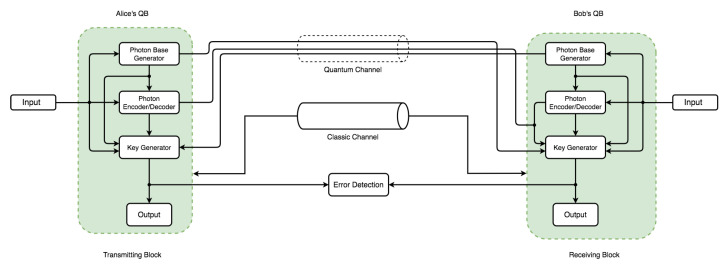
Simulation model design used for simulating an instance without the presence of eavesdropping (Eve’s quantum block).

**Figure 6:**
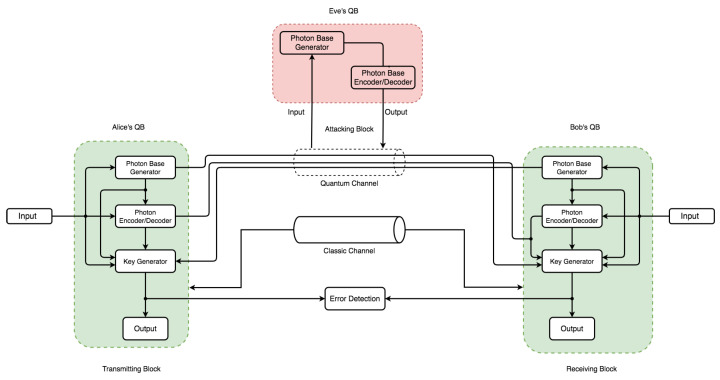
Simulation model design used for simulating an instance with the presence of eavesdropping (Eve’s quantum block).

The communication architecture model in Figure 5 and Figure 6 consists of an independent component within each QB with specific code base functionalities. Each QB has a photon-based generator ($PG_{b}$) and photon-based encoder/decoder ($PE/D_{b}$) component. However, only Parties A and B have a key generator ($KG_{b}$) with an output. Two channels operate on different principles: a quantum channel (security based on the laws of nature) and a classic channel (security based on mathematical and computational complexity). The flow and pattern assume Eve’s QB can intercept and re-transmit over the quantum channel via intercept-resend attack. This assumption only holds in discussing eavesdropping and its effect on the channel—the error between communicating parties handled via the error detection block. Because Eve’s presence requires both a photon-based generator and a photon-based encoder/decoder to perform both re-sending and interception operations, the two arrows leading to the quantum channel in Figure 6 denote that. Bitstreams from Eve’s operation can be sent to the receiver via the quantum channel, again with a major advantage for the comparison, effect, and verification of the principal concept that QKD can detect eavesdropping (Eve’s quantum block) transmission tampering.

### 4.2. Implementation

**Figure 7:**
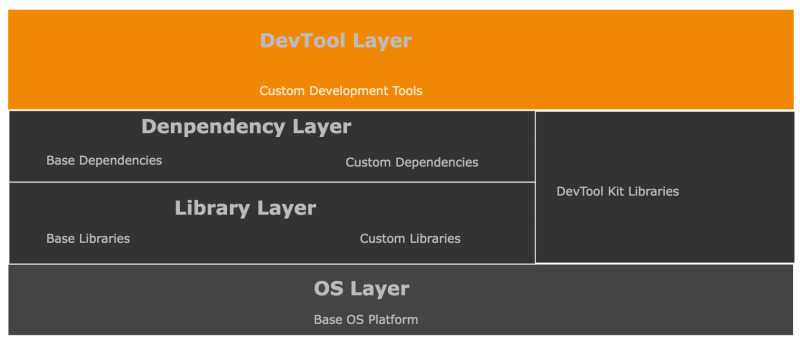
Simulation code base development environment.

This implementation uses a Linux environment with a significant advantage in its code base and implementation flexibility. It allows the utilization of open-source software libraries and modules. This simulation of the communication architecture model design uses a custom Python3 code base environment as shown in Figure 7. This figure represents the setup configuration of the simulation and development environment. It shows a stack of layers constituting our simulation requirements. The main language framework sits on top of the base operating system (OS) in the base library, while all external language core modules are base dependencies. The custom libraries and dependencies represent the simulation code for this implementation. Each code structure for the classes, functions, and packages follows the same naming convention used in both Figure 5 and Figure 6 to ensure consistency in code flow. For example, a $PG_{b}$ in Alice’s QB would be a single class with subsequent operations divided into functions converted into a custom package. There were two distinct simulation approaches used. The first instance was run without Eve’s block as the normal mode of operation in Figure 5, while the second instance in Figure 6 considered the presence of Eve’s QB. The algorithms for this implementation are in Algorithm 1.
**Algorithm 1** Custom code simulation using the BB84 protocol.1:**procedure** Simulation Procedure2:    **label:** *top*.3:    LBRgen←LowerboundrangeofbitRandom4:    HBRgen←HigherboundrangeofbitRandom5:    $\mathrm{Base}\leftarrow \mathrm{Base}\;\mathrm{Base}-\mathrm{MID}\;\mathrm{gen}\;-\;(0.5x10^{10})\;$6:    **if** Base>LBR gen **then** **return** |0〉7:    **end if**8:    **if** Base<HBR gen **then** **return** |1〉9:    **end if**10:    Assign a polarization base for each iteration of bit11:    Assign a polarization state for each iteration of bit12:    Compare each parties’ generated bit, polarization base, and polarization state13:    Calculate mismatch rate, error correction rate, error detection rate, and total error rate14:    **if** Errorrate>error threshold **then**15:        **goto** *top*.16:    **else**17:        Strengthen the final shared key via privacy amplification18:    **end if**19:    Final shared key is ready20:**end procedure**

The initial bitstream generation detection occurs during the simulation to know the exact amount of quantum bases for the encoding process. The generation of each bit sent to $PG_{b}$ undergoes a series of steps. The first step is assessing and evaluating the feeds to know precisely the needed single-photon bases to generate. It randomly assigns a quantum base for each bit separately, either horizontally biased or orthogonally biased. This step is the set polarization base class, which calls a random choice selection on a list containing the quantum base. Each time the set polarization step runs, the bit present at that particular instance is randomly assigned a base. The second step stores output bases iteratively from the previous step in each instance because the results need to reach $KG_{b}$.

In the third step (random polarization), each polarized bit from the first step corresponds to a single and specific quantum state (↑→↖↗) through a series of decision-making patterns. First, this step checks the polarization bases of the bit and the bit representation agreement between the communication parties beforehand. These parameters determine which quantum state needs assignment for each specific bit and polarization base. The results in this step stay in storage for replication and retrieval. However, the sender side uses $PE_{b}$, while the receiver uses the photon-based decoder ($PD_{b}$) and vice versa. $PG_{b}$ ensures the generation of the corresponding polarization base for the initial series of bits. $PE_{b}$ continues the step by evaluating the quantum bases from $PG_{b}$ to encode the bits. This stage checks if the polarization base matches the quantum state of each bit of generated information from Step 1 in $PG_{b}$ and then outputs the corresponding bit accordingly. To ensure that the data $PE_{b}$ utilized are precisely from the right source, the method responsible for this operation performs the length, data type, element validation, and assertions before and during code execution.

In a nutshell, the sender and receiver agree on bits that will represent the four quantum states. Afterward, Party A generates random streams of bits and feeds them to the QB to undergo quantum operations. The results are then sent to the receiver (Party B) in the initial stage using a quantum channel. The QB on Party B’s side also performs certain quantum operations and outputs the results based on B’s measurement criteria. B establishes communication on a classic channel, telling A the polarization bases of the measurement. Party A informs Party B on the same classic channel; the polarization bases are on the single-photon pulses sent. Both the sender and receiver share each other’s information without revealing any sensitive information on the classic channel. The exact process can be conversely bi-directional, where the receiver becomes the sender and vice versa. Parties A and B compare the stream of bits with each other’s information on the classic channel. The results then become the quantum key if both keys on both sides are equal. The process will start again if the bit error rate exceeds the acceptable threshold value for the QKD communication process.

The $KG_{b}$ section of the code implementation takes care of the data filtration and rectification processes. $KG_{b}$’s responsibility is to compare the sender and receiver data to generate the actual key in both halves after taking care of the data filtration and rectification processes. It compares the sender’s and receiver’s polarization base, measurement base, and stream of bits at each side along with the quantum states. The error detection component of this implementation evaluates the deviations within the streams of bits interchanged between the sender and the receiver. The process ensures the generation of the overall extraction of the quantum shared key. This component includes privacy amplification and other operations relating to the final quantum key.

## 5. Results and Discussions

The purpose of both simulation scenarios was to give a proof-of-concept of QKD based on the uncertainty and no-cloning principle, showing the advantages of using quantum cryptography for securing Internet communications, platforms, and infrastructures. The results are given in sections regarding the simulation steps and the processes involved. Each subsequent section presents the result in that simulation stage and discusses its significance.

### 5.1. Communication Phase Results

**Figure 8:**
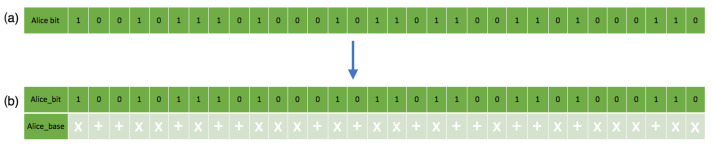
This figure shows bitstreams’ generation in the simulation: (**a**) shows the sender’s random bitstream. (**b**) shows the sender’s random bitstream with the associated polarization states.

The simulation models in both Figure 5 and Figure 6 illustrate two communication channels representing class objects. An agreement over a classic channel on the parameters in Table 1 between the sender and receiver occurs. The literature and theoretical study of QKD simulation in practice are affected by error correction [65] factors related to transmission errors, attacks, improper diode pulse configurations, time shifts, imperfect measurements, and other aspects of the overall quantum errors. As a result, the uncertainty accuracy in QKD research simulations ranges from 90 to 99 percent. However, in this work, even though the error threshold with Eve’s presence exceeds the threshold, 0.11 was chosen as the error threshold, consistent with the theory and literature [7]. They both do so without revealing any important information. As a result, the sender generates bitstreams and the associated quantum base output in Figure 8 using lower (0.0) and upper (1.0) limits with a precision of ten digits with a bit probability of 0.5. This limit allows the generation of bitstreams for base generation of 50 percent probability of either |0〉 or |1〉 per sample instance.

**Figure 9:**
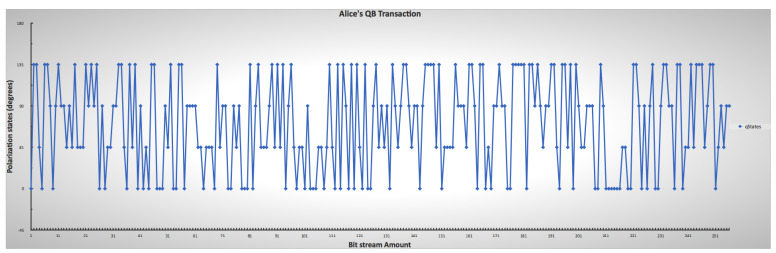
Sender’s lower and upper limit ranges for mimicking the chosen photon encoding through polarization, indicating the randomness of Alice’s choice.

**Figure 10:**
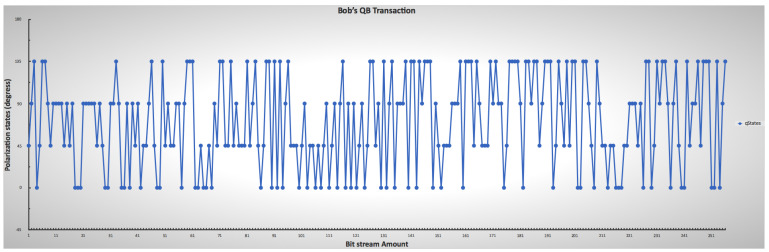
Receiver’s lower and upper limit ranges for mimicking the chosen photon encoding through polarization, indicating the randomness of Bob’s choice.

**Figure 11:**
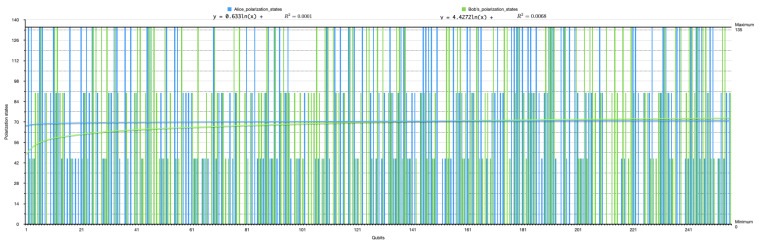
A histogram plot of the sender and receiver in the first simulation instance’s (without the presence of Eve) polarization state, indicating the chosen and measured qubits’ randomness.

Figure 9 and Figure 10 show the randomness of the sender’s and receiver’s QB qubit generation process for each bitstream, while Figure 11 shows the combination of both qubit generations. This approach mimics the condition in a real photon generator, which is modifiable to produce a desirable single photon. The initial base parameters and values influenced the operation and results of the custom code throughout the simulation.

**Figure 12:**
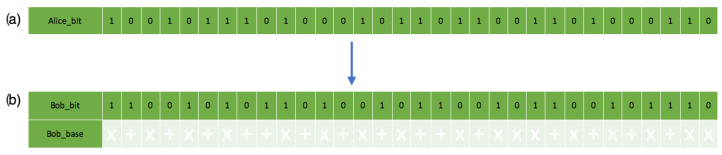
This is a figure showing the chosen bitstreams’ comparison: (**a**) Description of the sender’s chosen bitstreams. (**b**) Description of the receiver’s measurement and chosen bitstream with the associated polarization states.

On the receiver’s side, the recorded bit and polarizations through measurement sent from the sender in Figure 12 also show their matching polarization states. Figure 12b does not match Figure 8 because of quantum mechanics principles (no-cloning and Heisenberg’s uncertainty principle). The receiver measuring all single-photon pulses causes a disturbance, hence the changes in quantum states. The receiver also assumes a random quantum base and polarization quantum state, which reflect the bases below the bit in Figure 8b with the randomness in Figure 10.

### 5.2. Reconciliation Phase Results

**Figure 13:**
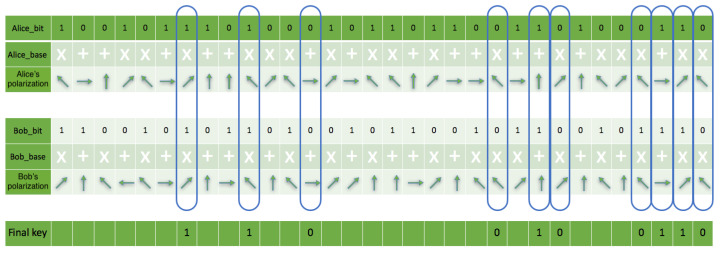
This is a figure showing a sample of the sender and receiver comparing each other’s selected results.

Comparing the implementation code and simulation result through the assertion technique ensures each node can identify channel-specific operations in multiple quantum nodes. The quantum measurement bases sent by both parties do not include their corresponding quantum states. The sender and receiver cross-check each other’s measurements by eliminating non-matching bases to produce the final result, as shown in Figure 13. Both parties produced approximately 0.546875 mismatches based on base retrieval in the communication channel during the simulation with an eavesdropping rate of 0.04296875 with an error correction rate of 0.2421875. However, it made no significant difference in the simulation re-iterations to the final secret shared key. The total number of bits (shared key) present on both sides after the reconciliation process was 116 out of 256 initial qubits.

### 5.3. Detection of Eavesdropper

**Figure 14:**
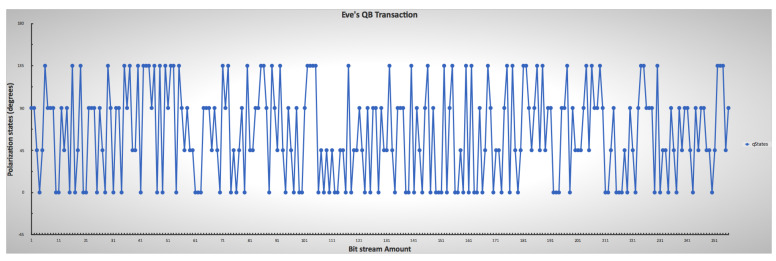
Eve’s lower and upper limit ranges for mimicking photon measurement and chosen encoding through polarization, indicating the randomness of Eve’s guesses.

**Figure 15:**
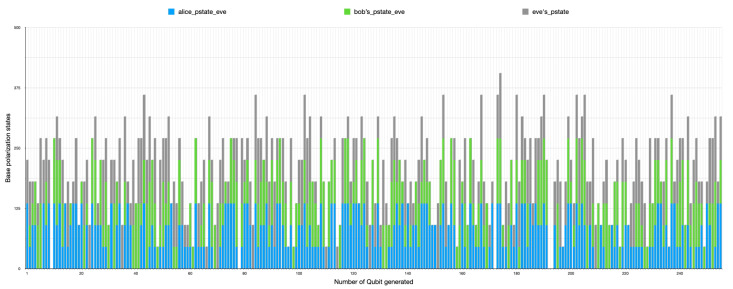
A 2D stacked bar chart with the sender’s, receiver’s, and Eve’s polarization states in the second simulation instance (with the presence of Eve), indicating the chosen and measured qubit randomness.

**Figure 16:**
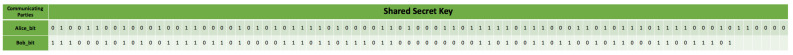
Sender and receiver final shared key in the reconciliation phase with the eavesdropper’s presence.

**Figure 17:**
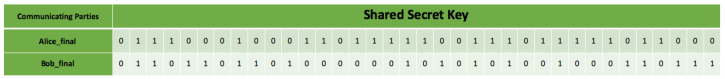
Sender and receiver final shared key mismatch with the eavesdropper’s presence.

Two types of error-checking operations took place during the simulation. The first error operation relates to each side’s qubit error during transmission as transmission errors during communication due to transmission factors such as noise, heat, environmental conditions, and others. The second part of the error process detects eavesdropping on the communication between parties by comparing the sub-keys. It takes a random sample of a specific length selected from the shared keys. A checking process then occurs by comparing if the base matches the initial stream of bits and sent bases for error detection. However, the errors attributed in the simulation by eavesdropping and the transmission processes are considered the same. Therefore, the total errors cannot be greater than the error threshold in Table 1. Figure 14 also shows the randomness of Eve’s guessing and chosen measurement during the transmission intercept-resend attack manipulation, while Figure 15 shows all combinations of all parties with the presence of Eve. The results show that an error correction rate of 0.265625 resulted in a variable shared key length out of the initial bits after a base-mismatch, as shown in Figure 16. There was a significant difference in the eavesdropper rate of 0.125 with a total key mismatch of a length of 36 after privacy amplification, shown in Figure 17. Table 2 lists some of the essential values relating to both simulations on the communication architectural model implementation.

In Figure 16, the size/length of the two bitstreams is different before privacy amplification because of the presence of Eve. Eve is intercepting the qubit on the quantum channel via intercept-resend attack, either detectable or not, depending on the key length. In this re-iteration simulation, Eve’s guess due to chance and probability was not favorable such that the altered and measured qubits by Bob influenced the initial shared key before privacy amplification, visible in the length/size.

### 5.4. Privacy Amplification Operations

The operation continues from the detection stage, intending to clean up the information leakage over the channel during the communication operations. The presence of an eavesdropper in a channel attack ensures that the probability of Eve making the right guess is 1/4. Hence, a simple privacy amplification process takes two separate random bits for an XOR operation to reduce the probability. The total number of keys left after privacy amplification is 54 out of 80 in the detection operation. Both sides compare their results, and if their results are the same, the shared key from detection is left untouched; if not, the bit elimination occurs at that specific index.

## 6. Conclusions

In this work, the communication architecture model and implementation of QKD using the BB84 protocol were presented. This communication architecture focused on the key distribution in python and eavesdropper detection utilizing quantum cryptography to enable secure communication through a proof-of-principle on quantum mechanics laws. The implementation of the model was carried out by developing a python custom base code with its efficiency analyzed through a series of aggregative simulation re-iterations in two scenarios. The communication architecture extended our simulation via modeling to allow the comparison of the initial and final parameters and the overall simulation modeling effect. We then carried out base reconciliation, rectification, and error correction operations on photon transmissions, consistent with the literature and theoretical results.

The first scenario is without the presence of an eavesdropper. It produced an eavesdropper rate of 0.04296875 since all error attributions were cumulative (including transmission, imperfect measurement, and others), with an error correction rate of 0.2421875 and a qubit probability of 0.5. This significantly led to a 54 bit-length shared key.

The second is with the eavesdropper’s presence through a methodological interception-resend attack from a practical perspective. Its results demonstrated the possibility of intercepting a quantum transmission via a quantum channel attack introducing a disturbance. However, such activities are detectable via the error rate attributed to transmission and eavesdropping. These rates resulted in a variable shared key length. Hence, the final shared key did not match due to an eavesdropper rate of 0.125 and an error correction rate of 0.265625, significantly more than the initial error threshold with a qubit probability of 0.5.

In some cases, the base-mismatch process discarded about 50 percent of the pre-initial shared key with an error threshold of 0.11 percent. However, it made no significant difference in the simulation re-iterations.

Future works can revolve around the power consumption analysis of the quantum cryptographic process and its deployment in resource-constrained devices without external QKD nodes, but embedded QKD nodes with a significantly improved error correction process.

## Figures and Tables

**Table 1:** Initial parameter used in the simulation.

Parameters	Values
Qubit length (bits)	256
Sender’s bit probability	0.5
Receiver’s bit probability	0.5
Attacker’s bit probability	0.5
Error threshold	0.11
Error detection sample length (bits)	128

**Table 2:** Comparison of base parameters used and obtained results from the two main simulation instances.

Parameters	Normal	Eavesdropping
Initial bits (bits)	256	256
Final key length (bits)	54	36
Error correction rate	0.2421875	0.265625
Eavesdropper rate	0.04296875	0.125
Party A, B bit probability	0.5	0.5
Eve bit probability	0.5	0.5
Base-mismatch (**%**)	0.546875	0.5234375

## Abbreviations

The following abbreviations are used in this manuscript:

IoT	Internet of Things
V2I	Vehicle-to-Internet
QKD	Quantum key distribution
BB84	Charles Bennett and Gilles Brassard’s protocol
QB	Quantum box
PG${}_{b}$	Photon-based generator
PE/D${}_{b}$	Photon-based encoder/decoder
PE${}_{b}$	Photon-based encoder
KG${}_{b}$	Key-based generator
PD${}_{b}$	Photon-based decoder
